# 1D Narrow-Bandgap Tin Oxide Materials: Systematic High-Resolution TEM and Raman Analysis

**DOI:** 10.3390/ma16134539

**Published:** 2023-06-23

**Authors:** Kazuhiro Manseki, Saeid Vafaei, Loren Scott, Katelyn Hampton, Nagisa Hattori, Kosuke Ohira, Kyle Prochotsky, Stephen Jala, Takashi Sugiura

**Affiliations:** 1 Graduate School of Natural Science and Technology, Gifu University, Yanagido 1-1, Gifu 501-1193, Japan; kmanseki@gifu-u.ac.jp (K.M.); a4524054@edu.gifu-u.ac.jp (N.H.);; 2 Mechanical Engineering Department, Bradley University, 1501 West Bradley Avenue, Peoria, IL 61625, USA; 3 Industrial and Manufacturing Engineering and Technology Department, Bradley University, Peoria, IL 61625, USA

**Keywords:** tin oxide, calcination, TEM, Raman, bandgap

## Abstract

We demonstrate for the first time the structure identification and narrow-bandgap property of 1D hybridized SnO/SnO_2_ nanoparticles derived from the calcination of a single-source precursor, i.e., tin(II) oxalate. Systematic Raman analysis together with high-resolution TEM (HR-TEM) measurements of the tin oxide samples were carried out by changing the calcination temperatures. These data revealed the simultaneous formation of 1D SnO/SnO_2_ in the rod particles that grew in air. It was also found that Sn(II) can be introduced by changing the concentration of Sn(II) salt in the precursor synthesis and the maximum temperature in calcination. Particles measuring 20~30 nm were sintered to produce tin oxide nanorods including tin monoxide, SnO. Photoabsorption properties associated with the formation of the SnO/SnO_2_ nanocomposites were also investigated. Tauc plots indicate that the obtained tin oxide samples had a lower bandgap of 2.9~3.0 eV originating from SnO in addition to a higher bandgap of around 3.5~3.7 eV commonly observed for SnO_2_. Such 1D SnO_x_/SnO_2_ hybrids via tin oxalate synthesis with this optical property would benefit new materials design for photoenergy conversion systems, such as photocatalysts.

## 1. Introduction

Tin oxide (SnO_2_) nanoparticles have a wide range of applications in transparent conductive electrodes, chemical gas sensors [1], lithium-ion batteries [2], solar-driven water splitting [3], and solar cells [4] because of their high electron mobility, optical transparency, excellent photostability, and because they do not require high sintering temperatures during production. The functionality of nanostructured metal oxide semiconductor materials has been studied extensively by exploiting the tunability of structure and property through chemical synthesis. Among the various reactions, a wet-approach, including sol–gel reaction and a hydrothermal process, is widely used in both academic and industrial fields [5,6]. The precise dimension control of nanomaterials ranging from 1D to 3D has been recognized as a key design criterion that can optimize the functionality of the materials.

For example, 1D nanostructured TiO_2_ with wire-shaped, tube-shaped, and rod-shaped morphologies has been utilized as an efficient electron transport material for photovoltaic applications, such as perovskite solar cells [7,8,9]. This is an intriguing property which affects understanding of the relationship between the structures of nanomaterials versus function. This concept of dimension-controlled materials can be further extended to hierarchical composite materials that consist of more than two different compounds, such as catalysts for batteries [10,11].

In recent years, tin oxide nanocomposites (SnO/SnO_2_) have received much attention because of their potential applications as p-n junction photocatalysts due to their visible-light absorbing properties [12]. Regarding photocatalysts of tin oxides, SnO_2−x_ nanocrystals with oxygen vacancies have also been designed for introducing defect centers, as well as for controlling band structures to improve their reactivities. Liu et al. have recently demonstrated that rich oxygen vacancy can be introduced in SnO_2_ nanocrystals for applications in visible-light photocatalysts [13]. Self-doping with Sn(II) was controlled by a hydrothermal method while changing the ratio of Sn(IV)/Sn(II) using SnCl_4_ and SnCl_2_ precursors. Nanocomposites, i.e., ZnO/SnO_2−x_ and SnO_2−x_/graphene oxide, have also been developed for investigating their photocatalytic activities [14,15]. For the synthesis of ZnO/SnO_2−x_, a simple hydrothermal approach using an SnCl_2_ precursor (SnCl_2_ dihydrate) was applied to produce SnO_2−x_, while SnCl_2_ dihydrate was also utilized in combination with Sn powder for hydrothermal reactions for SnO_2−x_/graphene oxide. Several tin oxide nanocomposites (SnO/SnO_2_) have also attracted increasing attention as gas sensors and in battery applications [16,17,18,19].

On the other hand, the reported photofunctional materials have often been limited to those with particulate-like morphologies. Therefore, the dimension control mentioned above could provide strategies to improve their functions. Although many reports have been published on SnO_2_ nanoparticles prepared by, as above, hydrothermal techniques and other methods, including low-temperature sol–gel reactions, the synthesis of anisotropic SnO_2_ nanoparticles and/or their film preparation, specifically the formation of a 1D morphology, has been a challenging topic. The traditional methods for producing 1D nanostructures are facile routes, such as virus templating, biomolecular-assisted synthesis, and solution-phase precursors [20].

In earlier research, Y. Wang et al. demonstrated the potential of polymer-directed synthesis of SnO_2_ nanowires for gas-sensing applications [21]. They reported on a solution-based precursor method using tin(II) oxalate as a starting material, followed by calcination to remove all organic compounds and form porous 1D SnO_2_ nanowires. Furthermore, several studies have been made on the analogous synthesis of 1D SnO_2_ by modifying the template polymer and reaction temperatures [22]. Using this synthetic approach, D. Hu et al. successfully demonstrated the formation of high-surface area SnO_2_ nanorods and their performance as gas-sensing materials [23].

**Figure 1:**
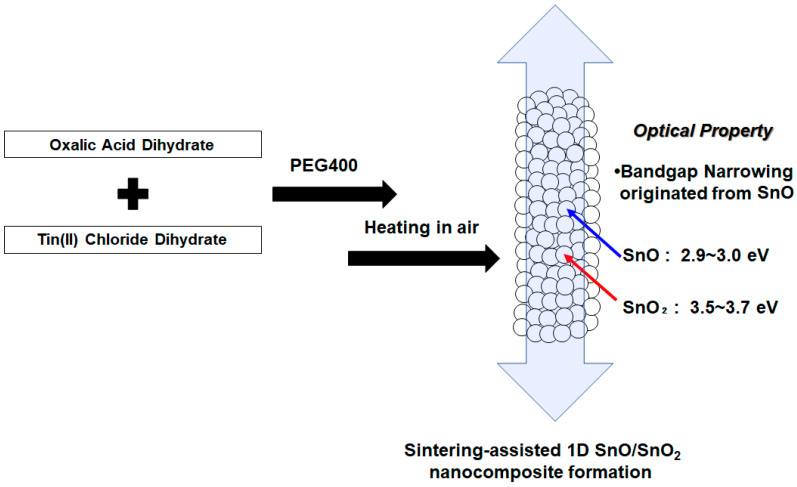
Conceptual drawing of the temperature-controlled isolation of SnO/SnO_2_ powder samples via PEG-based sol–gel and heating approaches. Optical properties of the hybrid materials originating from the formation of SnO is also presented.

As of now, no reports on the bandgap engineering of 1D-shaped tin oxides are available. This is partially because of the wide-bandgap property of SnO_2_ (3.6 eV) [24]. In terms of photoenergy conversion that requires broader light absorption as well as the efficient photocarrier transportation of electrons and holes, as in photocatalysts, our focus is unveiling the formation of lower-bandgap SnO using the template approach with tin oxalate precursor in 1D SnO/SnO_2_ nanoparticle experiments, as illustrated in Figure 1. We demonstrate the optical properties of 1D SnO/SnO_2_, including photoabsorption that shows a narrowed bandgap (2.9~3.0 eV). Sn(II) can be introduced by the conversion of the precursor under calcination in air when the condition of Sn(II) complexation in polyethylene glycol and the sintering strategy are adjusted. The structure characterization of 1D SnO/SnO_2_ using HR-TEM measurements and Raman spectroscopy, as well as their photoabsorption properties, will be elucidated.

## 2. Materials and Methods

### 2.1. Chemicals

Tin chloride dihydrate (SnCl_2_·2H_2_O), oxalic acid dihydrate, and polyethylene glycol (PEG, approximate molecular weight 400) were purchased from Fujifilm Wako Chemicals, Osaka, Japan and used as received. Ethanol (99.5%) was also purchased from Kanto Chemical Co., Inc., Tokyo, Japan. H_2_O (resistivity: 18.2 MΩ·cm) was obtained using a Milli-Q^®^ integral water purification system (MERCK Ltd., Tokyo, Japan).

### 2.2. Synthesis of SnO/SnO_2_ Nanorods

We controlled the tin salt concentration and complexation temperature as follows. Typically, 0.39 g of oxalic acid dihydrate was dissolved in a mixed solvent of 3 mL ethanol and 3 mL of PEG, and the mixture was kept at 0 °C or 60 °C. Then, 0.11 g of tin chloride dihydrate was added, and 1 mL of water was added dropwise while maintaining the given temperature to produce white precipitates.

For comparison, 0.06 g or 0.22 g of tin chloride dehydrate was also reacted in a similar way to perform the above reaction, where 0.20 g or 0.78 g of oxalic acid was used to synthesize the tin oxalate, respectively. After stirring the solution for 5 min, centrifugation (10,000 rpm) was performed for 15 min. The supernatant was removed, and the precipitate was centrifuged with water (10,000 rpm) for 5 min. After the supernatant was discarded, ethanol was added. The precipitation was further treated with centrifugation (10,000 rpm) for 5 min. The resulting samples were then dried and calcined at 350 °C, 400 °C, 450 °C, 500 °C, and 600 °C in air to produce powder samples. The period of maximum temperature was set to 1.5 h unless otherwise noted. The ramping rate to reach the maximum temperature was 16 °C/min. An electric muffle furnace (FUW 232PA, ADVANTEC, Osaka, Japan) was used for the calcination.

### 2.3. Characterization of SnO/SnO_2_ Nanorods

The synthesized powder samples were characterized using X-ray diffraction (XRD; Rigaku RINT Ultima/PC with monochromated Cu–Kα radiation, Tokyo, Japan). Crystallite size of the obtained particles was estimated using the Scherrer equation (D = Kλ/βcosθ), where D, K, λ, and θ indicate crystallite size, Scherrer constant (0.90), X-ray wavelength (1.54 Å), and Bragg angle, respectively. The tin oxide samples were analyzed using XPS (XPS; ULVAC, Quantera SXM, Chigasaki, Japan). Nanostructures were characterized using SEM (HITACHI S-4800, Tokyo, Japan) and TEM (JEM-2100, Tokyo, Japan). Ultraviolet–visible (UV–Vis) spectra were obtained from the diffuse reflectance of the powder samples using a Hitachi U-4000 spectrophotometer. TG-DTA analysis in air flow was carried out using DTG 60H (SHIMADZU, Kyoto, Japan). The ramping rate was 10 °C/min. A confocal Raman microscope, RAMANtouch (Nanophoton Corp., Tokyo, Japan), was used to analyze the tin oxide samples. The powder samples were irradiated with laser light of 532 nm, and the laser power was adjusted to 5 mW/cm^2^.

## 3. Results and Discussions

The polyethylene glycol (PEG)-template method was applied for synthesizing 1D tin oxalate for conversion into tin oxides. We anticipated that the amount of tin source in the precursor, i.e., the ratio of SnCl_2_ and oxalic acid, would affect the decomposition phenomena of the organic parts due to the different oxidation reactions of high valent Sn(IV) sources with increasing temperatures. We changed the concentration of tin chloride and the calcination temperature and time to produce tin oxides while maintaining the 1D morphology of the precursor, as discussed below. Reaction temperatures in the precursor synthesis and amount of tin source were 0 °C, 0.11 g; 60 °C, 0.06 g; 60 °C, 0.22 g; and (60 °C, 0.11 g).

**Figure 2:**
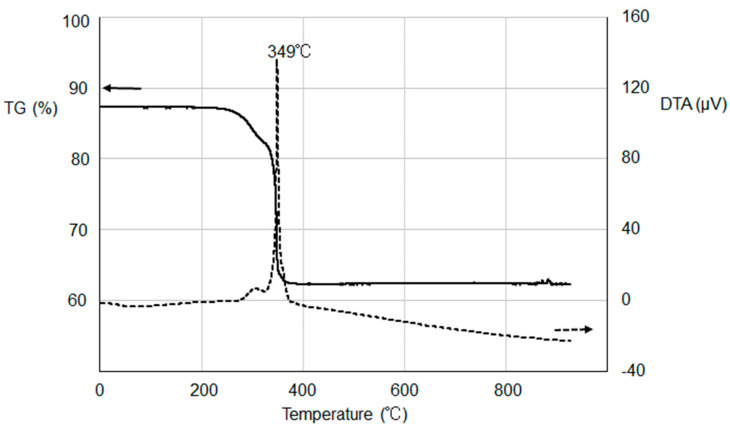
TG−DTA curves of the tin oxalate precursor measured in air.

Figure 2 shows TG-DTA curves of one of the tin oxalate precursors, which was prepared using the largest amount of Sn(II) chloride compound (0.22 g). We found from the TG curve that the loss of sample weight almost completed at around 350 °C. The DTA curve indicates a marked exothermic peak at 349 °C, suggesting the combustion of most of the organic parts. A noticeably lower decomposition temperature was observed for our precursor compared to those of 375 °C or higher in some studies [23,25]. This is probably due to highly efficient oxidation reactions in coexistence with an excess amount of Sn(II) to organic ligands.

**Figure 3:**
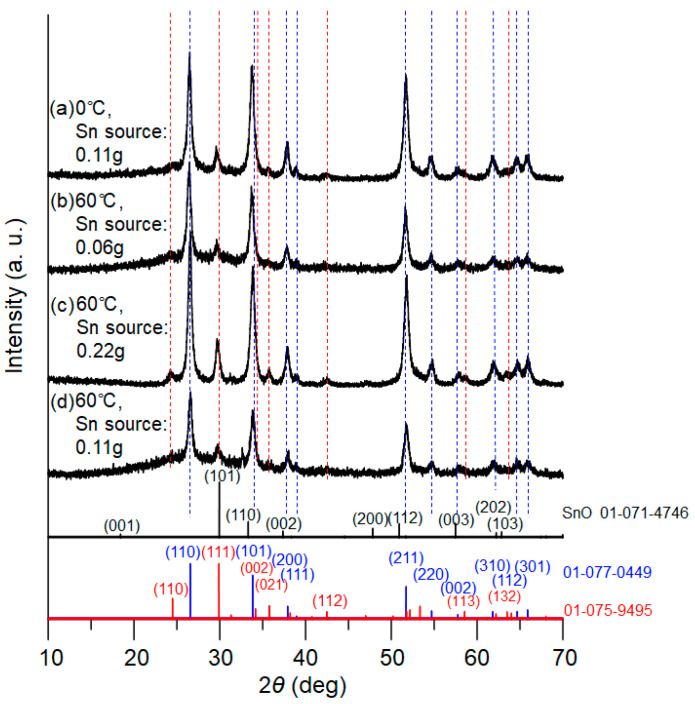
XRD patterns of the tin oxide samples calcined for 1.5 h at 500 °C. Reaction temperatures in the precursor synthesis and the amount of tin source: (**a**) 0 °C, 0.11 g; (**b**) 60 °C, 0.06 g; (**c**) 60 °C, 0.22 g; and (**d**) 60 °C, 0.11 g. JCPDS data of SnO, SnO_2_ (tetragonal), and SnO_2_ (orthorhombic) are presented at the bottom.

Nucleation control of the precursor compound, tin oxalate, was studied by systematically changing the reaction temperatures and concentrations of tin chloride, as described in the experimental section. Figure 3 presents XRD patterns of the powder samples after calcination for 1.5 h, for which the tin oxalate powders obtained at 0 °C and 60 °C in the PEG solution were utilized. For the 60 °C reactions, the concentration of metal source was also varied. The XRD patterns clearly indicate that both tetragonal and orthorhombic SnO_2_ simultaneously formed for all samples. Taking Figure 3c (with the larger amount of tin salt) as an example, diffraction peaks corresponding to the tetragonal phase of SnO_2_ were detected at 2ϴ = 26.6°, 33.8°, 37.9°, 38.9°, 51.7°, 54.7°, 57.8°, 62.0°, 64.6°, and 65.8°, whereas the peaks at 24.3°, 29.7°, 33.2°, 35.7°, 42.5°, 58.6°, and 63.3° were assigned to the orthorhombic phase. The XRD patterns match well with those of the database.

**Figure 4:**
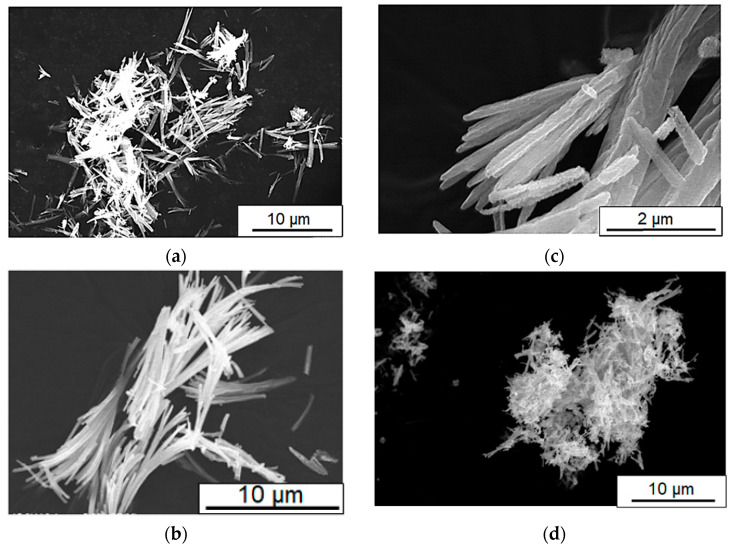
SEM images of the tin oxide sample with 1.5 h of calcination at 500 °C. Reaction temperatures in the precursor synthesis and the amount of tin source: (**a**) 0 °C, 0.11 g; (**b**) 60 °C, 0.06 g; (**c**) 60 °C, 0.22 g; and (**d**) 60 °C, 0.11 g.

Typical SEM images of the synthesized samples are displayed in Figure 4. The aspect ratio of the short and long axis of the 1D-shaped tin oxides were found to be around 1:20~1:30 for the 0 °C and 60 °C reaction samples (tin source = 0.06 g) (Figure 4a,b). On the other hand, the aspect ratio significantly decreased to around 1:10 for the other 60 °C reaction samples (tin source = 0.11 g and 0.22 g) (Figure 4c,d). It is most likely from the observed scale of 1D growth that nucleation of the tin oxalate precursor was promoted at the higher temperature of 60 °C, and a case can also be made for increased concentrations of tin chloride, leading to the formation of a shorter rod-shaped morphology.

**Figure 5:**
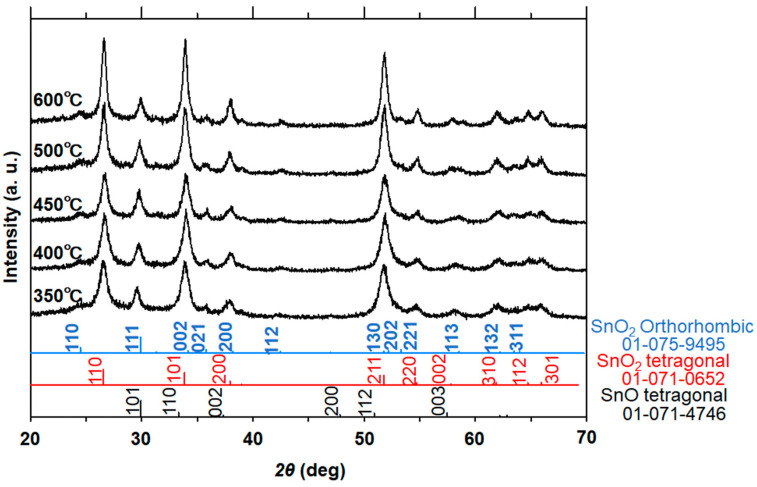
XRD patterns of the tin oxide samples calcined for 1.5 h at different temperatures. The maximum temperatures of each calcination condition were 350 °C, 400 °C, 450 °C, 500 °C, and 600 °C. JCPDS data of SnO, SnO_2_ (tetragonal), and SnO_2_ (orthorhombic) are also shown. Reaction temperature in the tin oxalate precursor synthesis and amount of tin source were 60 °C and 0.22 g, respectively.

**Figure 6:**
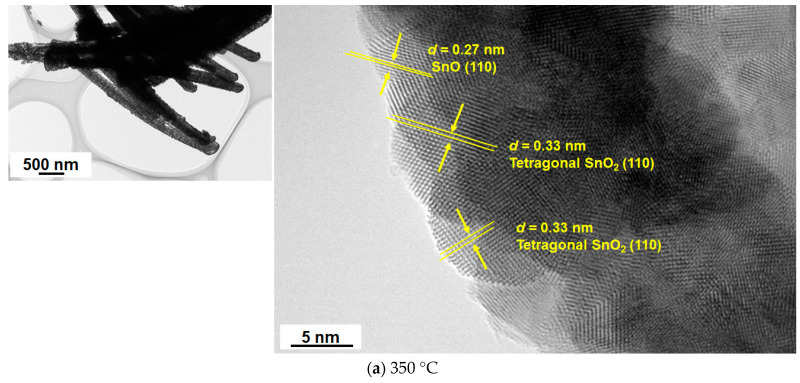

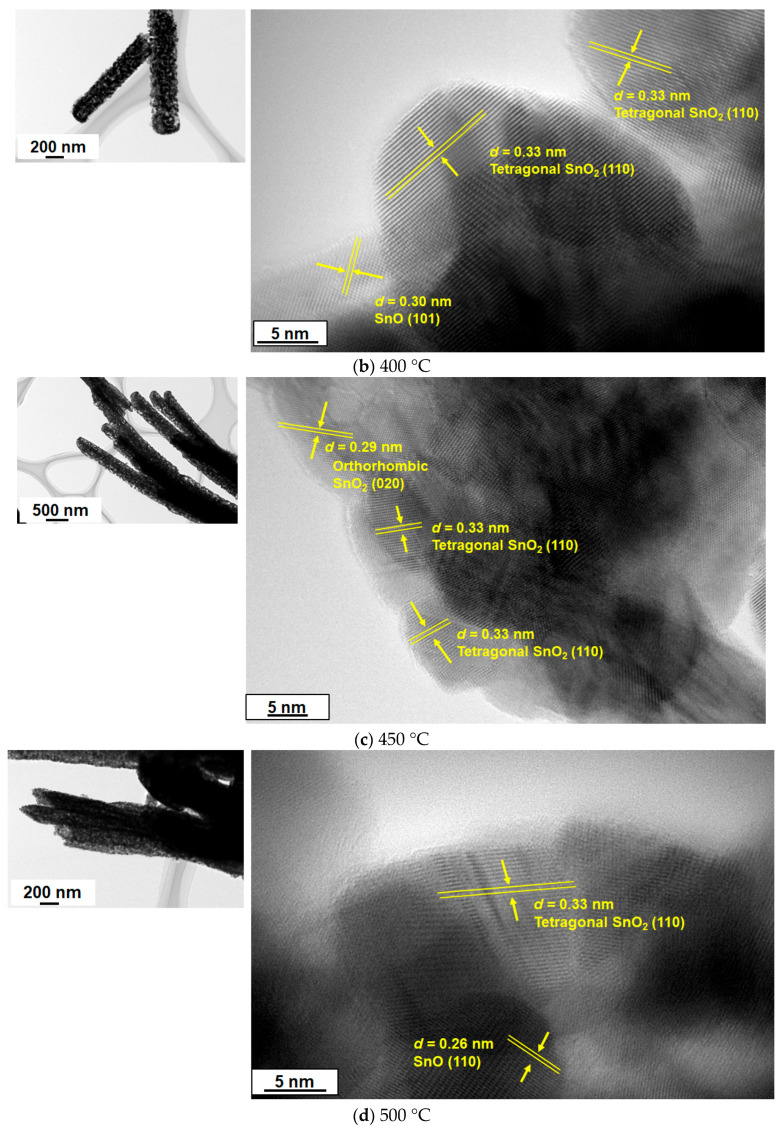

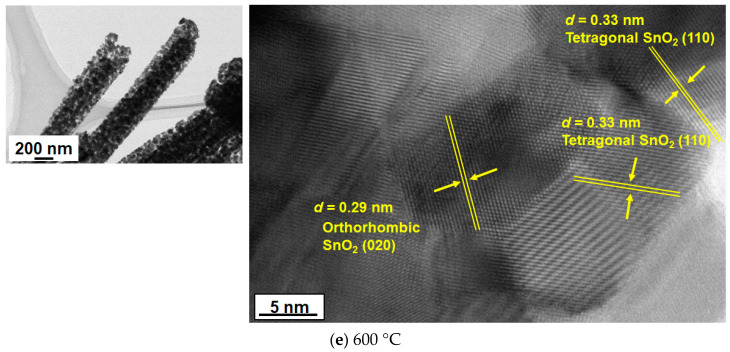
Low- and high-resolution TEM images of the tin oxide samples calcined at (**a**) 350 °C, (**b**) 400 °C, (**c**) 450 °C, (**d**) 500 °C, and (**e**) 600 °C. Reaction temperatures in the precursor synthesis and amount of tin chloride were 60 °C and 0.22 g, respectively.

**Figure 7:**
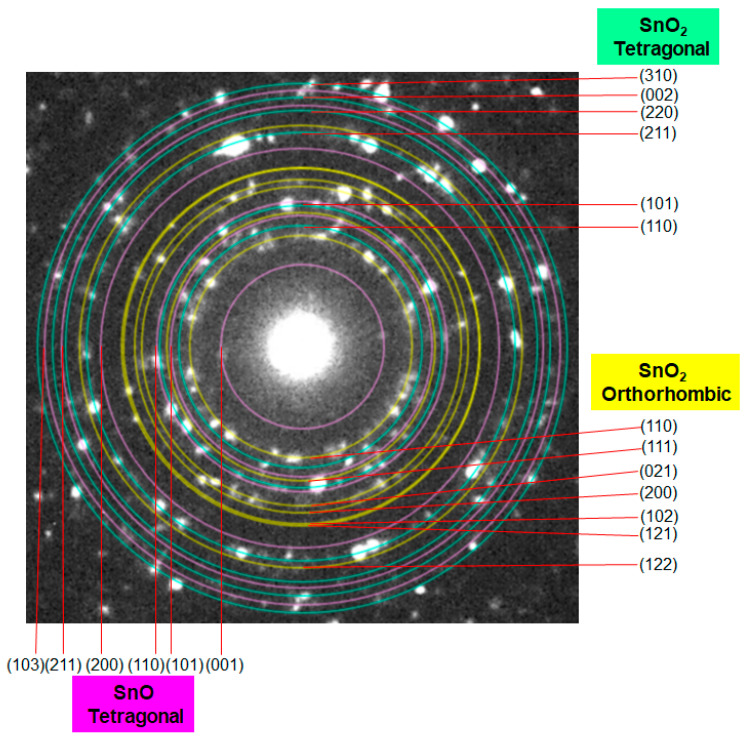
An example selected area diffraction (SAD) pattern of the TEM measurement of the 500 °C sample calcined for 1.5 h.

The 1D nanostructure formation for the sample in Figure 4c was also confirmed by TEM measurement, as discussed below. We analyzed the XRD and TEM images of the samples calcined at 350 °C, 400 °C, 450 °C, 500 °C, and 600 °C (Figure 5). Similar to the data in Figure 3, the XRD patterns in Figure 5 indicate the simultaneous formation of both tetragonal and orthorhombic SnO_2_ for all samples. Importantly, the HR-TEM analysis allowed us to understand the SnO phase formation that was not detected in the XRD patterns because of the difficulty of analyzing the major diffraction peaks of SnO that are almost the same as those of orthorhombic SnO_2_, e.g., at 29.9° (Figure 3 and Figure 5). In terms of the existence of SnO, Figure 6 clearly indicates lattice fringes of *d* = 0.27 nm and 0.30 nm, which correspond to the (110) and (101) planes of SnO, respectively. A selected area diffraction (SAD) pattern of the 500 °C sample is depicted in Figure 7, where the observed spots have been identified as those of the three structure phases (tetragonal SnO_2_, orthorhombic SnO_2_, and tetragonal SnO). The SAD pattern also indicates the polycrystalline nature of the 1D sample consisting of several primary nanoparticles.

**Figure 8:**
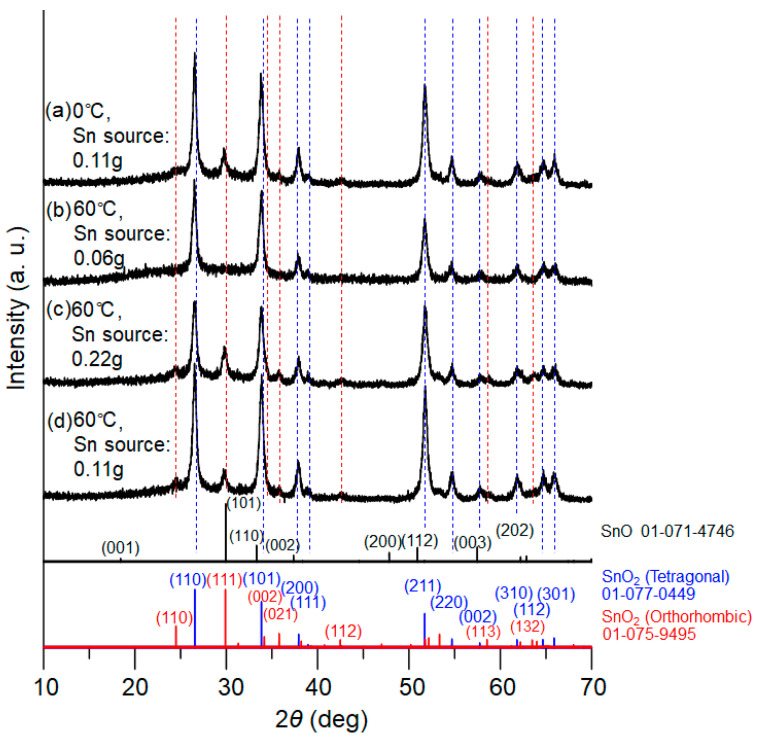
XRD patterns of the tin oxide samples calcined for 6 h at 500 °C. Reaction temperatures in the precursor synthesis and the amount of tin source: (**a**) 0 °C, 0.11 g; (**b**) 60 °C, 0.06 g; (**c**) 60 °C, 0.22 g; and (**d**) 60 °C, 0.11 g. JCPDS data of SnO, SnO_2_ (tetragonal), and SnO_2_ (orthorhombic) are also shown.

Using the four samples described in Figure 3, we also examined 6 h of calcination while keeping the maximum temperature at 500 °C. The XRD patterns are presented in Figure 8. It can be observed in Figure 8b that the sample using a smaller amount of tin salt exhibits the disappearance of all orthorhombic phase peaks, whereas the peaks of interest for other conditions remain. In contrast to the yellowish-gray color for most of the powder samples (1.5 h calcination), it becomes white in Figure 8b (6 h calcination). Such a difference was observed only for this reaction condition (tin source = 0.06 g). Therefore, it is suggested that the PEG-assisted oxidation of SnO, as well as the orthorhombic to tetragonal phase transition, are accelerated during calcination when the relative tin concentration to PEG is lower.

From the XRD data (Figure 5), the estimated crystallite sizes with respect to the (110) plane of tetragonal SnO_2_ increased with increasing calcination temperatures (9 nm for 350 °C, 13 nm for 450 °C, and 21 nm for 600 °C) in 1.5 h of calcination. Taking the samples in Figure 3c and Figure 8c as examples, the calculated crystallite sizes for orthorhombic SnO_2_, corresponding to (021) planes, increased from 20 nm to 28 nm with longer calcination, suggesting enhanced crystallization in the nanorod formation. As shown in Figure 6, typical lattice fringes found in the nanoparticles were assigned to tetragonal SnO_2_ (*d* = 0.33 nm), orthorhombic SnO_2_ (*d* = 0.29 nm), and tetragonal SnO (*d* = 0.26 nm and 0.30 nm). It should be noted that the formation of SnO in the polymer-directed calcination approach has been evidenced here for the first time by TEM analysis. Apparently, the concentration of tin chloride, the maximum temperature of calcination, and the period of maximum temperature are the key factors that determine nanocomposite formation of 1D SnO/SnO_2_. A reduced state of Sn(0) or other tin oxide species such as Sn_2_O_3_ and Sn_3_O_4_ was not detected in our synthesis, suggesting that the SnO was converted directly into SnO_2_. One of the plausible mechanisms of tin oxides is that the oxidation reaction of Sn(II) hydroxide species in the early stages of calcination (1.5 h) produces SnO as a mixture of SnO/orthorhombic SnO_2_/tetragonal SnO_2_.

The authors of [23] point out that the SnO precursor plays an important role in the formation of orthorhombic SnO_2_ during calcination, although they did not isolate the SnO-containing samples in their synthesis. This conversion mechanism from SnO to orthorhombic SnO_2_ is consistent with our new HR-TEM data of the isolated powder sample presenting the concomitant formation of SnO and orthorhombic SnO_2_, as shown in Figure 6.

**Figure 9:**
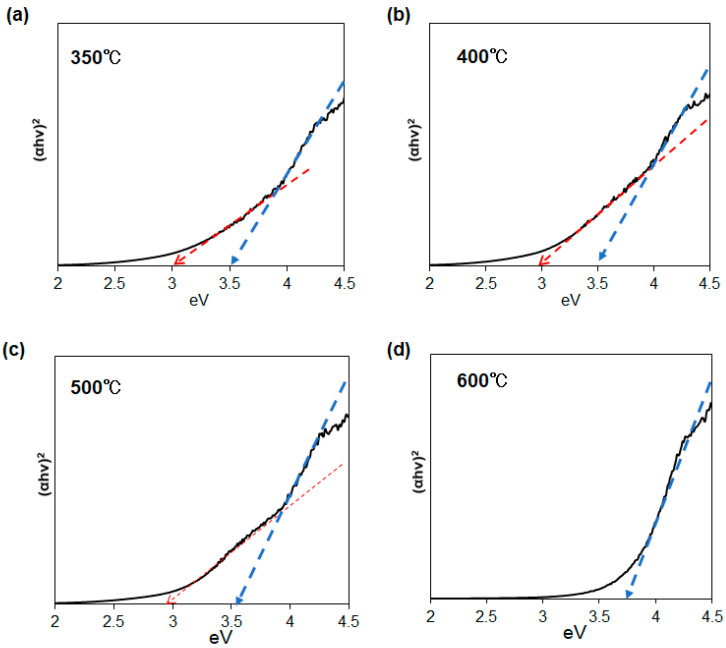
Tauc plots of the tin oxide samples calcined for 1.5 h at different temperatures. The maximum temperatures of each calcination condition were (**a**) 350 °C, (**b**) 400 °C, (**c**) 500 °C, and (**d**) 600 °C. The red and blue dotted lines correspond to a lower and higher bandgap, respectively.

The optical properties of the SnO/SnO_2_ nanoparticles were elucidated not only from absorption spectra but also XPS analysis. Tauc plots of several SnO/SnO_2_ samples, obtained from diffuse reflectance spectroscopy, are presented in Figure 9. The bandgaps estimated from the Tauc plot are listed in Table 1. Except for the 600 °C sample, the results clearly indicate that our nanohybrid materials have two bandgaps at 2.9~3.0 eV and 3.5~3.7 eV. To the best of our knowledge, this is the first evidence of the formation of 1D narrow bandgap SnO/SnO_2_ nanoparticles using a single-source precursor approach.

The absorption spectra of several SnO/SnO_2_ samples calcined at 500 °C for 6 h were also measured. When the reaction temperatures in the precursor synthesis and the amount of tin source were 0 °C and 0.11 g, 60 °C and 0.06 g, 60 °C and 0.22 g, and 60 °C and 0.11 g, respectively, the lower bandgap was estimated to be 2.9~3.0 eV except for the largest amount of tin source (0.06 g). The narrower bandgaps observed in the calcination samples were similar to those of reported SnO bandgaps (2.8–3.0 eV) [12]. For longer calcination conditions, a marked increase in bandgap was clearly observed only for 0.06 g of tin source as a result of the enhanced oxidation of the tin oxides.

**Figure 10:**
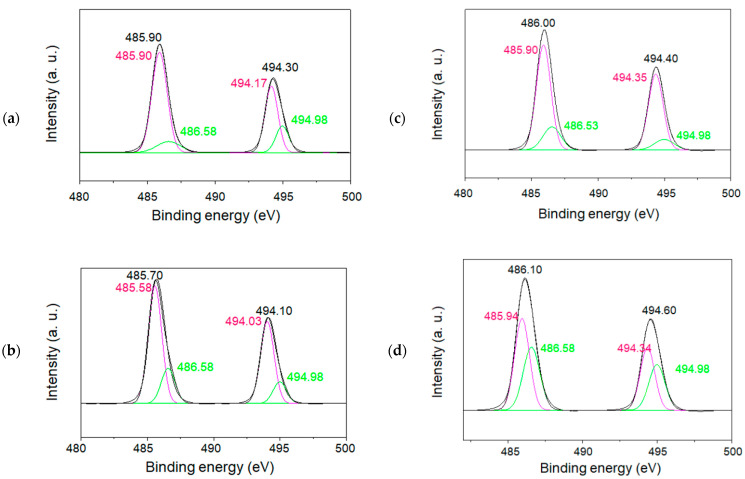
XPS spectra (Sn3d) of the tin oxide samples calcined for 1.5 h at 500 °C. The black lines are deconvoluted into two peaks with the pink and green lines. Reaction temperatures in the precursor synthesis and the amount of tin source: (**a**) 0 °C, 0.11 g; (**b**) 60 °C, 0.06 g; (**c**) 60 °C, 0.22 g; and (**d**) 60 °C, 0.11 g.

**Figure 11:**
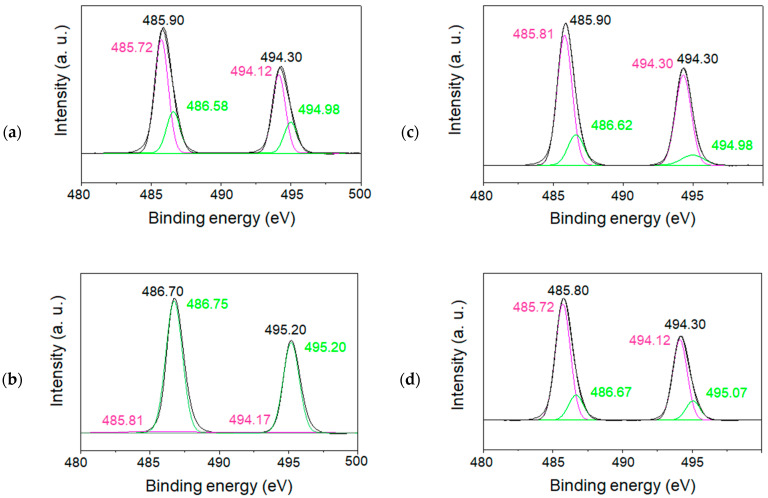
XPS spectra (Sn3d) of the tin oxide samples calcined for 6 h at 500 °C. The black lines are deconvoluted into two peaks with the pink and green lines. Reaction temperatures in the precursor synthesis and the amount of tin source: (**a**) 0 °C, 0.11 g; (**b**) 60 °C, 0.06 g; (**c**) 60 °C, 0.22 g; and (**d**) 60 °C, 0.11 g.

In order to characterize the charge state of tin ions in the SnO/SnO_2_ nanoparticles, XPS measurements for both the 1.5 and 6 h samples calcined at 500 °C were also performed, as illustrated in Figure 10a–d and Figure 11a–d. All spectra corresponding to the spin–orbit doublet peaks of Sn 3d3/2, and Sn 3d5/2 were deconvoluted into two peaks. The binding energies of the peaks at approximately 486 eV and 495 eV were assigned to Sn 3d5/2 and Sn 3d3/2, respectively [26,27]. Their XPS studies and others demonstrate that the formation of Sn(II) and Sn(IV) species can be identified from the decomposition of each Sn3d line. Based on the reported binding energies of 485.9 eV for Sn(II) and 486.6 eV for Sn(IV), the simulation of raw data indicated the formation of both Sn(II) and Sn(IV) in SnO/SnO_2_. For the samples calcinated for 6 h (Figure 11), sample (b) showed the formation of less Sn(II)-containing nanoparticles, which is also in agreement with the wide bandgap property mentioned above.

**Figure 12:**
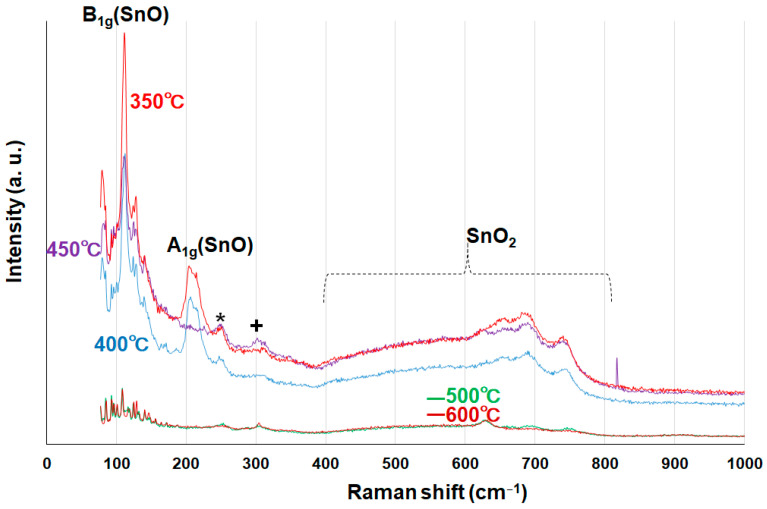
Raman spectra of the tin oxide powder samples calcined for 1.5 h at temperatures of 350 °C, 400 °C, 450 °C, 500 °C, and 600 °C. Reaction temperatures in the precursor synthesis and the amount of tin chloride were 60 °C and 0.22 g, respectively. The peaks (* and +) were intermediate compounds corresponding to Sn_3_O_4_ and Sn_2_O_3_, respectively.

To further prove the hybridization of SnO and SnO_2_ in the rod particles, systematic Raman analysis was performed using the tin oxide powder samples calcined for 1.5 h at temperatures of 350 °C, 400 °C, 450 °C, 500 °C, and 600 °C, as shown in Figure 12. It is well known that the vibrational modes of tin monoxide SnO can be distinguished from SnO_2_ modes, and the two modes of SnO (B_1g_ and A_1g_) are assigned to around 110 cm^−1^ and 208 cm^−1^, respectively [28]. We found that peaks corresponding to 110 cm^−1^ were clearly observed for all samples and that the intensities were relatively small for the 500 °C and 600 °C samples. The results suggest that SnO oxidation is enhanced for higher calcination times and/or temperatures, as discussed in relation to the XPS data. The vibrational modes of SnO_2_ were found in the range of 400~800 cm^−1^, allowing us to characterize SnO and SnO_2_ nanohybrids in the rod particles. It appears that the laser-induced formation from SnO to Sn_2_O_3_ and/or Sn_3_O_4_ took place during the Raman measurements [29] because of the oxidation reactions, taking into consideration that these intermediate species were not found in the HR-TEM and XRD data. In other words, this observation supports the concomitant formation of SnO and SnO_2_ in the rod particles.

## 4. Conclusions

For the first time, we have reported the identification of hybridized SnO/SnO_2_ nanoparticles with 1D morphology, mainly by systematic Raman analysis and HR-TEM measurement. The calcination temperatures of the tin oxalate precursor were adjusted and 1D SnO/SnO_2_ were obtained. Sn(II) formation was verified using not only HR-TEM measurements but also Raman and XPS measurements. A lower bandgap of 2.9~3.0 eV corresponding to absorption in the visible light region was estimated from diffuse reflectance spectra. The results of this study provide useful insights into the design of photofunctional tin oxide nanomaterials. One example of this is that this new synthesis can be extended to develop energy materials for visible-light absorbing photocatalysts.

## Figures and Tables

**Table 1:** Estimated bandgap energies of obtained SnO/SnO_2_ nanoparticles. Reaction temperature in the precursor synthesis and amount of tin source were 60 °C and 0.22 g, respectively.

Calcination Temperatures	Bandgaps
350 °C	3.0 eV, 3.5 eV
400 °C	3.0 eV, 3.5 eV
500 °C	2.9 eV, 3.5 eV
600 °C	3.7 eV

## Data Availability

Not applicable.
